# Treatment outcomes of patients with multidrug-resistant and extensively drug resistant tuberculosis in Hunan Province, China

**DOI:** 10.1186/s12879-017-2662-8

**Published:** 2017-08-16

**Authors:** Kefyalew Addis Alene, Hengzhong Yi, Kerri Viney, Emma S. McBryde, Kunyun Yang, Liqiong Bai, Darren J. Gray, Archie C. A. Clements, Zuhui Xu

**Affiliations:** 1 Research School of Population Health, College of Medicine, Biology and Environment, The Australian National University, Canberra, ACT Australia; 2 Institute of Public Health, College of Medicine and Health Sciences, University of Gondar, Gondar, Ethiopia; 3 Department of MDR-TB, Internal Medicine, Hunan Chest hospital, Changsha city, Hunan Province China; 4 Centre for Global Health, Department of Public Health Sciences, Karolinska Institutet, Stockholm, Sweden; 5 Australian Institute of Tropical Health and Medicine, James Cook University, Townsville, QLD Australia; 6 Department of Director’s Office, Tuberculosis Control Institute of Hunan Province, Changsha city, Hunan Province China; 7 Department of Tuberculosis Control, Tuberculosis Control Institute of Hunan Province, Changsha city, Hunan Province China

**Keywords:** Multidrug-resistant, Extensively drug resistant, Tuberculosis, Treatment outcomes, China

## Abstract

**Background:**

The worldwide emergence of multidrug-resistant tuberculosis (MDR-TB) and extensively drug-resistant tuberculosis (XDR-TB) has posed additional challenges for global tuberculosis (TB) control efforts, as limited treatment options are available and treatment outcomes are often sub-optimal. This study determined treatment outcomes among a cohort of MDR-TB and XDR-TB patients in Hunan Province, China, and identified factors associated with poor treatment outcomes.

**Methods:**

We conducted a retrospective study using data obtained from medical records of TB patients in Hunan Chest Hospital, and from the internet-based TB management information system managed by the Tuberculosis Control Institute of Hunan Province, for the period 2011 to 2014. Treatment outcomes were assessed for patients diagnosed with MDR-TB (TB resistant to at least isoniazid and rifampicin) and XDR-TB (MDR-TB plus resistance to any fluoroquinolone and at least 1 second-line injectable drug). Cumulative incidence functions were used to estimate time to events (i.e. poor treatment outcomes, loss to follow-up, and unfavourable treatment outcomes); and a competing-risks survival regression model was used to identify predictors of treatment outcomes.

**Result:**

Of 481 bacteriologically-confirmed patients, with a mean age of 40 years (standard deviation SD ± 13 years), 10 (2%) had XDR-TB and the remainder (471; 98%) had MDR-TB. For the entire cohort, treatment success was 57% (*n* = 275); 58% (*n* = 272) for MDR-TB and 30% (*n* = 3) for XDR-TB. Overall, 27% were lost to follow-up (*n* = 130), 27% (*n* = 126) for MDR-TB and 40% (*n* = 4) for XDR-TB; and 16% had a poor treatment outcome (*n* = 76), 15% for MDR-TB and 30% (*n* = 3) for XDR-TB. Of the 10 XDR-TB patients, 3 (30%) completed treatment, 3 (30%) died and 4 (40%) were lost to follow-up. Of the 471 MDR-TB patients, 258 (57%) were cured, 16 (3%) completed treatment, 13 (3%) died, 60 (13%) experienced treatment failure, and 126 (27%) were lost to follow-up. Resistance to ofloxacin was an independent predictor of poor (AHR = 3.1; 95%CI = 1.5, 6.3), and unfavourable (AHR = 1.7; 95%CI = 1.07, 2.9) treatment outcomes. Patients who started treatment during 2011–2012 (AHR = 2.8; 95% CI = 1.5, 5.3) and 2013 (AHR = 2.1; 95% CI = 1.2, 3.9) had poorer treatment outcomes compared to patients who started treatment during 2014.

**Conclusion:**

Patients with MDR-TB and XDR-TB had low rates of treatment success in Hunan Province, especially among patients who started treatment during 2011 to 2013, with evidence of improved treatment outcomes in 2014. Resistance to ofloxacin was an independent predictor of poor treatment outcomes.

## Background

Tuberculosis (TB) remains the largest infectious disease killers worldwide [1]. According to the World Health Organization (WHO), in 2015, there were an estimated 10.4 million new TB cases, 1.4 million TB deaths and another 0.4 million deaths among people with TB and HIV [2]. Multidrug-resistant tuberculosis (MDR-TB), defined as TB resistant to at least isoniazid and rifampicin, and extensively drug-resistant tuberculosis (XDR-TB), defined as MDR-TB plus resistance to any fluoroquinolone and at least 1 second-line injectable drug, have become major public health problems, and pose additional challenges for global TB control efforts [3, 4]. In 2015, there were an estimated 480,000 new cases of MDR-TB and an additional 100, 000 new cases of rifampicin resistant (RR) TB [2]. In the same year, there were 7579 new cases of XDR-TB reported from 74 countries [2]. Approximately 45% of the global MDR-TB burden occurs in China, India and the Russian Federation [2].

China is one of the top 30 MDR-TB and TB burden countries in the world [5]. According to WHO’s latest global TB report, there were an estimated 57,000 cases of MDR/RR-TB among notified pulmonary TB cases in China in 2015 [2]. This accounted for 12% of the total MDR-TB burden globally in 2015. A national MDR-TB survey, conducted in 2007 demonstrated that 5.7% of new cases and 25.6% of previously treated cases had MDR-TB, and approximately 8% of the patients with MDR-TB had XDR-TB [6].

The increasing number of people with MDR-TB and XDR-TB represents a threat to national TB control efforts [3], because the treatment of MDR-TB and XDR-TB takes longer than drug-susceptible TB, has toxic side effects and treatment outcomes are often poor. Ongoing transmission, failure to diagnose drug resistant TB at initial presentation, and high mortality and treatment dropout rates are also challenges [7, 8]. According to the WHO’s 2016 Global TB Report, in 2015, a total of 9, 662 MDR-TB and 357 XDR-TB patients were diagnosed and confirmed by laboratory investigations in China; of these, a total of 5691(58.9%) MDR-TB and 122 (34.2%) XDR-TB patients started second-line TB treatment [2]. The treatment success rates for people with MDR-TB and XDR-TB in 2013 were 55% and 22%, respectively [2]. These treatment success rates are below the WHO target of a 75% treatment success rate by the end of 2015 [9].

Information about treatment outcomes in patients with drug resistant-TB and factors associated with poor treatment outcomes is limited in China. Therefore, the aim of this study was to assess treatment outcomes and to identify factors associated with poor treatment outcomes and loss to follow-up in a cohort of HIV negative and culture-confirmed MDR-TB and XDR-TB patients in Hunan Province, China.

## Methods

### Study design and setting

A retrospective cohort study was conducted in Hunan Province, China. The province is located in central-south China and has a population of approximately 72 million people [10]. The average annual TB incidence rate in Hunan Province was 78.9 per 100,000 population during the period 2005–2009 [11]. The proportions of MDR- and XDR-TB among all TB patients were 10.6–25.2% and 1.8% [12, 13], higher than the national proportions [6].

Hunan Chest Hospital is the only chest hospital in the province. It is located in Changsha, the capital city of the province. The hospital has 610 beds, and provides diagnostic and treatment services for patients with chest and lung diseases including TB, MDR-TB, and XDR-TB, who are referred from throughout the province. In 2011, the hospital established an MDR-TB treatment centre which provides comprehensive diagnostic and treatment services for patients with drug resistant TB. The MDR TB treatment centre serves as a referral hospital for all persons with presumptive drug resistant TB in the province, although those with known HIV co-infection go to a different hospital.

### MDR- TB and XDR-TB diagnosis

Of the 131 counties in Hunan Province, 32 counties are able to provide comprehensive diagnostic services, which include culture. However, drug susceptibility testing (DST) is mainly carried out in the Hunan Chest Hospital. Thus, sputum specimens from all culture-positive TB patients from throughout the province are referred to the Hunan Chest Hospital for DST. In the hospital, phenotypic DST based on solid and liquid culture techniques, and molecular methods using line probe assays as well as Xpert® MTB/RIF are performed. At the Hunan Chest Hospital, DST is performed for rifampicin, isoniazid, ethambutol, streptomycin, kanamycin, and ofloxacin. For the initial diagnosis of MDR-TB, liquid cultures are used for 9 (1.9%) of cases and solid cultures are used for 472 (98.1%) of cases. Solid culture is also used for the follow-up of patient’s progress and treatment outcomes.

### Treatment regimen and management

Patients with bacteriologically confirmed MDR-TB and XDR-TB are admitted to the MDR-TB treatment centre at the Hunan Chest Hospital for treatment and management. Patients are treated with an individualized treatment regimen containing four drugs, based on their DST results and history of previous TB treatment. The regimen usually includes an injectable agent (i.e. kanamycin, amikacin or capreomycin), a fluoroquinolone (i.e. levofloxacin, ofloxacin or moxifloxacin), para-aminosalicylic acid, prothionamide, pyrazinamide, clarithromycin, ethambutol, or cycloserine. The duration of treatment is 24 months for patients with MDR-TB and 30 months for patients with XDR-TB. The injectable drugs are used for a minimum of 6 months for MDR-TB patients, and 12 months for XDR-TB patients. Patients are admitted to the hospital for one to 2 months during the intensive phase and while hospitalised, receive directly observed therapy (DOT) by trained medical staff. During this time, patients also receive psychological support and counselling from hospital nurses. When the patients are medically fit, they are treated as out-patients. They receive support from trained family members or from trained supervisors in the community and return to the hospital once a month for a drug refill. As part of routine care, sputum microscopy and cultures are performed monthly for the first 2 months, and thereafter every other month until the end of treatment.

### Data sources and variables

All MDR-TB and XDR-TB patients registered from the establishment of the MDR-TB treatment centre from 2011 to 2014 were included in this study. This period was purposely selected so that 24-month treatment outcomes could be assessed. Patients who were diagnosed with MDR-TB but who did not start treatment were excluded from the study. Patients who were transferred out and whose treatment outcomes were not assessed were also excluded.

Patient information including socio-demographic characteristics such age, sex, and occupation; clinical variables such as year treatment commenced, treatment outcomes, duration of therapy and previous TB treatment history; as well as smear, culture and DST results, were obtained from an internet-based TB Management Information System in the Tuberculosis Control Institute of Hunan Province, and from MDR-TB medical records and the DST registration book at Hunan Chest Hospital. Given that all TB and HIV co-infected patients are treated in a separate hospital, the cohort did not include information about HIV status and all patients included in this study were assumed to be HIV-negative. Information obtained from the different data sources was linked using the patient’s registration number.

### Treatment outcomes

Treatment outcomes are assigned by a team of physicians working in the hospital based on the patient’s progress (i.e. based on adherence to treatment and signs of clinical improvement), and culture results. In the surveillance system, treatment outcomes are recorded as cured, treatment completed, died, treatment failure (failure due to side effects, or failure due to other reasons), lost to follow-up (i.e. default) or not evaluated (others). These treatment outcomes are based on WHO recommendations [14]. Treatment outcomes were defined as follows:

- **Cured** was defined as someone who completed treatment without evidence of treatment failure and who had three or more consecutive negative cultures taken at least 30 days apart, after the intensive phase.
- **Treatment completed** was defined as a patient who had completed treatment but did not meet the definition for cured due to a lack of bacteriological results.
- **Treatment failure** was defined as treatment terminated or a need for permanent regimen change of at least two anti-TB drugs due to an adverse drug reaction, or lack of culture conversion by the end of the intensive phase, or bacteriological reversion in the continuation phase after conversion to negative after intensive phase, or evidence of additional acquired resistance to fluoroquinolones or second-line injectable drugs.
- **Lost to follow-up** was defined as a patient whose treatment was interrupted for two consecutive months or more.
- **Death** was defined as those who died for any reason during the course of treatment.

Treatment outcome, the dependent variable of this study, was categorized into two mutually exclusive groups: 1) treatment success if the patient was cured or completed treatment; and 2) unfavourable outcomes if the patient died, had an outcome of treatment failure, or was lost to follow-up. For further analysis, unfavourable treatment outcomes were separated into two groups: 1) lost to follow-up if the patients were “lost” during treatment; and 2) poor treatment outcome if the patient died or had an outcome of treatment failure.

### Data analysis

We undertook descriptive analyses, calculating means with standard deviations (SD) for normally distributed continuous variables, medians with an interquartile range (IQR) for non-normally distributed continuous variables and percentages for categorical variables. In addition, we used Fisher’s exact test for comparison of categorical variables and the Wilcoxon rank sum test for the comparison of ordinal or continuous measures.

Cumulative incidence functions were used to estimate time to events (i.e. time to poor treatment outcomes, time to loss to follow-up, and time to unfavourable outcomes); and a competing-risks survival regression model was used to identify predictors of treatment outcomes. The analysis included all drug-resistant cases (i.e. both MDR-TB and XDR-TB cases).

Time to treatment outcomes was measured in months from the start of MDR/XDR-TB treatment to the occurrence of the outcome event. In the analysis, successful treatment outcomes (i.e. cured or treatment completed) were considered as censored. Three different events were considered separately to analyze the different type of treatment outcomes individually and to see their composite outcomes. The first event was time to unfavourable treatment outcomes (i.e. poor treatment outcome, or loss to follow up, whichever came first) and it was a composite of the second and the third events. The second event was time to loss to follow up, and the third event was time to poor treatment outcome (i.e. death or treatment failure, whichever came first). In the time to poor treatment outcome analysis, loss to follow-up was considered as a competing risk (i.e. events that occur instead of the event of interest). This computing event (i.e. loss to follow up) cannot be treated as censored because it might be dependent on poor treatment outcome that could not be measured using the current data. Similarly, in the time to loss to follow-up analysis, death was considered as a competing risk. In the presence of competing events, it is recommended to use cause-specific hazards rather than standard hazards, and cumulative incidence estimates rather than the Kaplan-Meier survival estimates [15]. Therefore, we used competing-risks survival regression models, instead of Cox regression models, to identify the predictors of poor treatment outcomes and loss to follow-up. Variables with statistical significance at *p* < 0.2 in the bivariate analysis, and variables with clinical significance (i.e. irrespective of their statistical significance) based on the literature, were fitted in the final multivariate analysis. Crude and adjusted hazard ratios with their 95% CI intervals were calculated to measure time to treatment outcomes. The analysis were performed using STATA version 14.1 [16].

## Results

### Demographic characteristics

A total of 493 patients were registered with a diagnosis of MDR-TB or XDR-TB during 2011–2014. Four patients had not commenced treatment after a diagnosis of MDR-TB and eight patients whose treatment outcome was not recorded were excluded from the study. Thus, the remaining 481 patients (471 MDR-TB and 10 XDR-TB) were eligible and were included in the analysis. The majority of patients were farmers (380; 79%) and male (340; 71%). The mean age of the patients was 40 years (SD ±13 years). Table 1 summarizes the demographic and clinical characteristics of patients.

**Table 1:** Demographic and clinical characteristics of patients with multidrug-resistant tuberculosis and extensively drug-resistant tuberculosis in Hunan Province, China, from 2011 to 2014

Characteristics	Total *n* = 481	MDR-TB^a^ *n* = 471	XDR-TB^b^ *n* = 10	*P*-value*
Age in years, mean ± SD^a^	40.4 ± 12.9	40.4 ± 12.9	40.6 ± 11.3	0.85
Male gender, n (%)	340 (70.7)	331 (70.3)	9 (90.0)	0.29
Occupation, n (%)				0.11
Farmer	380 (79.0)	374 (79.4)	6 (60.0)	
Labourer	15 (3.1)		−	
Employed	16 (3.3)	15 (3.2)	−	
Unemployed	19 (3.8)	16 (3.4)	−	
Other/unknown	51 (10.6)	19 (4.0) 47 (10.0)	4 (40.0)	
Year treatment commenced, n (%)				0.10
2011	2 (0.4)	2 (0.4)	−	
2012	118 (24.5)	116 (24.2)	4 (40.0)	
2013	164 (34.1)	159 (33.7)	5 (50.0)	
2014	197 (40.9)	196 (41.6)	1 (10.0)	
Positive sputum smear result at commencement, n (%)	477 (99.1)	467 (99.1)	10 (100.0)	0.91
Previous TB or MDR-TB treatment, n (%)	417 (86.7)	408 (86.6)	9 (90.0)	0.60
Resistant number of drugs, median (IQR)	3 (2–4)	3 (2–4)	5 (5–6)	0.05
Resistant to Ethambutol (E), n (%)	165 (34.3)	161 (34.2)	4 (40.0)	0.80
Resistant to Streptomycin (S), n (%)	288 (59.9)	279 (59.2)	9 (90.0)	0.19
Resistant to Ofloxacin (Ofx), n (%)	46 (9.6)	37 (7.9)	9 (90.0)	<0.001
Resistant to Kanamycin (Km), n (%)	15 (3.1)	6 (1.3)	9 (90.0)	<0.001
Treatment outcome, n (%)				0.14
Cured	261 (54.3)	258 (54.8)	3 (30.0)	
Completed	14 (2.9)	14 (2.9)	−	
Successful treatment (cured + completed)	275 (57.2)	272 (57.8)	3 (30.0)	
Died	13 (2.7)	13 (2.8)	−	
Failure	63 (13.1)	60 (12.7)	3 (30.0)	
Poor treatment outcome (death + failure)	76 (15.8)	73 (15.5)	3 (30.0)	
Lost to follow up	130 (27.0)	126 (26.8)	4 (40.0)	
Unfavourable outcome (died + failure +lost to follow-up)	206 (42.8%)	199 (42.3)	7 (70)	
Duration of therapy in months, median (IQR^c^)	24 (13–25)	24 (13–25)	22.5 (14–26)	0.87
Sputum smear conversion, n (%)	414 (86.1)	404 (85.8)	10 (100)	0.37
Culture conversion, n (%)	386 (80.2)	380 (80.7)	6 (60.0)	0.11
Time of sputum smear conversion in months, median (IQR^c^)	1(1–2)	1(1–2)	1.5 (1–8)	0.37
Time of culture conversation in months, median (IQR^c^)	1 (1–3)	1 (1–2)	5 (1–20)	0.03

^a^ *MDR-TB* multidrug resistant tuberculosis, *SD* standard deviation, ^b^XDR-TB, extensively drug-resistant tuberculosis

^c^ *IQR* inter quartile range

*Comparison between MDR-TB and XDR-TB patients calculated with Wilcoxon rank-sum test for continuous variables and Fisher’s exact test for categorical variables

### Clinical characteristics

The prevalence of XDR-TB among all culture-confirmed MDR-TB was 2% (95% CI: 1% -4%). The majority of MDR-TB (408; 87%) and XDR-TB (9; 90%) patients had been previously treated for TB or MDR-TB. Of the 417 previously treated patients, 8 (2%) patients were previously treated with both first-line and second-line anti-TB drugs, and 409 (98%) patients were treated with first-line anti-TB drugs only. The median time for culture conversion was higher in XDR-TB patients at 5 months (IQR 1–20 months) than in MDR-TB patients at 1 month (IQR: 1–2 months) (*p* = 0.03). The duration of hospitalization was recorded for 321 patients. The median length of hospital stay for these patients was 23 days (IQR = 18–31 days); 23 days (IQR = 18–31 days) for MDR-TB patients and 22 days (IQR = 20–29 days) for XDR-TB patients. The median hospital stay was similar for patients with a successful treatment outcome (21 days; IQR 17–31 days), a poor treatment outcome (23 days; IQR18–30 days) and loss to follow-up (24.5 days; IQR 18–32 days).

### Treatment outcomes

Of all 481 MDR-TB and XDR-TB patients, 262 (54%) were cured, 14 (3%) completed treatment, 13 (3%) died, 63 (13%) had treatment failure and 130 (27%) were lost to follow-up. Of 10 patients with XDR-TB, 3 (30%) were cured, 3 (30%) had treatment failure, and 4 (40%) were lost to follow-up. The overall treatment success rate for all patients was 57% (95% CI: 52%–61%), 58% for patients with MDR-TB (*n* = 272) and 30% for patients with XDR-TB (*n* = 3) (Table 1). Overall, 76 (21%) patients had poor treatment outcomes (13 patients died and 63 patients had treatment failure). The proportion of poor treatment outcomes did not differ when comparing those with or without previous anti-TB treatment; 70 (17%) versus 6 (9%) among patients without and with a history of previous TB treatment, respectively (*p* = 0.2; Table 2).

**Table 2:** Treatment outcomes of multidrug-resistant tuberculosis and extensively drug-resistant tuberculosis patients stratified by previous anti-TB treatment in Hunan Province, China, from 2011 to 2014

Treatment outcome	No. (%) patients previously treated, *n* = 417	No. (%) patients not previously treated, *n* = 64	*P*-value
Cured	228 (54.7)	33 (51.6)	0.20
Completed	11 (2.6)	3 (4.7)	
Successful treatment (cured + completed)	239 (57.3)	36 (56.3)	
Died	11 (2.6)	2 (3.1)	
Failure	59 (14.2)	4 (6.2)	
Poor treatment outcome (death + failure)	70 (16.8)	6 (9.4)	
Lost to follow up	108 (25.9)	22 (34.4)	

### Survival analysis of all MDR/XDR-TB patients

**Fig. 1:**
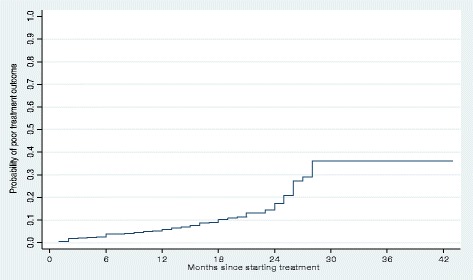
Cumulative hazard estimate of the probability of a poor treatment outcome (i.e. death or failure) in patients with multidrug-resistant tuberculosis and extensively drug-resistant tuberculosis in Hunan Province, China, 2011–2014

**Fig. 2:**
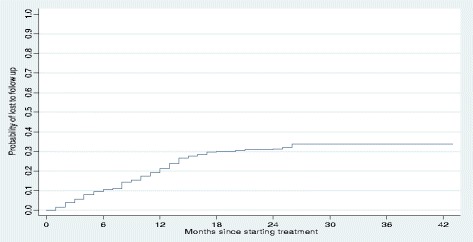
Cumulative hazard estimate of the probability of loss to follow-up in patients with multidrug-resistant tuberculosis and extensively drug-resistant tuberculosis in Hunan province, China, 2011–2014

**Fig. 3:**
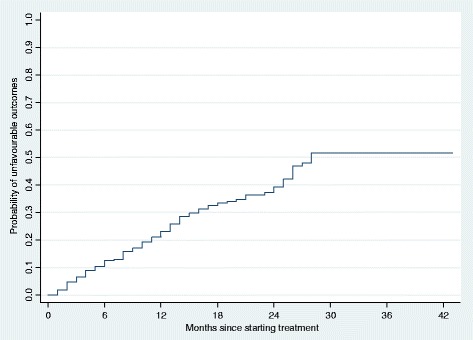
Cumulative hazard estimate of the probability of an unfavorable treatment outcome (i.e. death, treatment failure or loss to follow-up) in patients with multidrug-resistant tuberculosis and extensively drug-resistant tuberculosis in Hunan Province, China, 2011–2014

More than two thirds (77%) of poor treatment outcomes occurred during the continuation phase (i.e. after 6 months of therapy). For all patients, the cumulative probability of a poor treatment outcome at the end of 6 months was 5%, at the end of 1 year was 7%, and at the end of 2 years was 18% (Fig. 1). Patients with an outcome of lost to follow-up interrupted treatment after a median of 10 months of treatment (IQR: 5–14 months); two thirds (87/130 or 67%) occurred during the continuation phase of treatment. The cumulative probability of loss to follow-up at the end of 6 months was 9%, at the end of 1 year was 21%, and at the end of 2 years was 31% (Fig. 2). The overall cumulative probability of unfavorable outcomes (i.e. death, treatment failure or loss to follow up combined) was 12% at the end of 6 months, 23% at the end of 1 year, and 40% at end of 2 years (Fig. 3). The median hazard time or the time at which the cumulative hazard function of an unfavourable outcome in all patients was equal to 0.5, was 27 months (Fig. 3).

**Fig. 4:**
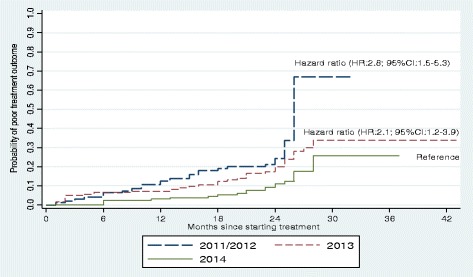
Cumulative hazard estimate of the probability of poor treatment outcome (i.e. death or treatment failure) in patients with multidrug-resistant tuberculosis and extensively drug-resistant tuberculosis by year of initiation of treatment in Hunan Province, China, 2011–2014

Patients who started treatment in 2011–2012 and 2013 had shorter survival times than patients who started treatment in 2014. The cumulative probabilities of a poor treatment outcome at the end of 1 year and 2 years were 13% and 27% for patients who started treatment in 2011–2012, 7% and 20% for patients who started treatment in 2013, and 3% and 11% for patients who started treatment in 2014, respectively (Fig. 4).

### Predictors of poor treatment outcome and lost to follow-up

Resistance to ofloxacin was an independent predictor of poor (AHR = 3.1; 95%CI = 1.5, 6.3), and unfavourable (AHR = 1.7; 95%CI = 1.07, 2.9) treatment outcomes. Patients who started treatment during 2013 had an almost two times higher risk of poor treatment outcomes compared to patients who started treatment during 2014 (AHR = 2.1; 95% CI = 1.2, 3.9). Moreover, patients who started treatment during 2011–2012 had an almost three times higher risk of poor treatment outcomes compared to patients who started treatment during 2014 (AHR = 2.8; 95% CI = 1.5, 5.3). When we compared those who were lost to follow-up to those with a successful treatment outcome, no statistically significant hazard ratio was observed (Table 3).

**Table 3:** Predictors of poor treatment outcome (i.e. death or failure), loss to follow-up, and unfavourable outcomes (i.e. death, treatment failure, or loss to follow-up) in patients with multidrug-resistant and extensively drug-resistant tuberculosis in Hunan Province, China, 2011–2014

Variables	Poor treatment outcome		Lost to follow up		Unfavourable outcome	
	CHR^b^ (95% CI)	AHR^c^ (95% CI)	CHR^b^ (95% CI)	AHR^c^ (95% CI)	CHR^b^ (95% CI)	AHR^c^ (95% CI)
Age in years	1.0 (0.9–1.0)	1.0 (0.9–1.01)	1.01(1.0–1.03)	1.0 (0.9–1.02)	1.01 (1.0–1.02)	1.01 (1.0–1.02
Male gender	1.2 (0.7–2.0)	1.1 (0.7–1.8)	1.4 (0.9–2.2)	1.4 (0.9–2.1)	1.4 (1.04–2.0)	1.3 (0.97–1.9)
Occupation^a^						
Labourer	0.5 (0.1–3.1)	0.3 (0.06–2.1)	1.6 (0 .7–3.8)	1.9 (0.8–4.3)	1.2 (0.6–2.7)	1.3 (0.6–2.8)
Employed	0.4 (0.1–3.0)	0.4 (0.05–3.2)	0.6 (0.2–2.0)	0.7 (0.2–2.4)	0.5 (0.2–1.4)	0.6 (0.2–1.5)
Unemployed	2.0 (0.7–4.9)	1.8 (0.7–4.8)	1.1 (0.5–2.)	1.4 (0.6–3.2)	1.5 (0.8–2.7)	1.7 (0.9–3.2)
Other/unknown	1.5 (0.8–2.8)	1.4 (0.7–2.6)	0.9 (0.5–1.6)	1.01 (0.5–1.8)	1.0 (0 .7–1.6)	1.1 (0.7–1.8)
Treatment starting year^a^						
2011–2012	2.8 (1.5–5.2)	2.8 (1.4–5.0)	0 .6 (0.4–1.1)	0.6 (0.3–1.03)	1.1 (0 .7–1.5)	1.0 (0.7–1.5)
2013	2.1 (1.2–3.8)	2.0 (1.1–3.8)	0 .7 (0 .5–1.1)	0.7 (0.5–1.1)	1.0 (0.7–1.4)	0.9 (0.7–1.4)
Previous anti-TB treatment	1.8 (0.8–4.2)	1.8 (0.8–4.2)	0 .7 (0.5–1.2)	0.8 (0.5–1.2)	0.9 (0.6–1.4)	0.9 (0.6–1.4)
Resistant to Ethambutol	1.2 (0.8–2.0)	1.3 (0.8–2.1)	1.1 (0.7–1.5)	1.1 (0.7–1.6)	1.1 (0.8–1.5)	1.2 (0.9–1.6)
Resistant to second-line injectable drug	1.3 (0.8–2.2)	1.3 (0.8–2.1)	0.7 (0 .4–1.0)	0.7 (0.5–1.02)	0 .8 (0.6–1.1)	1.7 (0.5–5.4)
Resistant to Ofloxacin	2.8 (1.4–5.6)	3.1 (1.5–6.3)	0.9 (0.4–1.8)	1.01 (0.5–2.1)	1.5 (0.9–2.5)	1.7 (1.07–2.9)

^a^For occupation farmer, and for treatment starting year 2014 was the referent category

^b^CHR, crude hazard ratio; ^c^AHR, adjusted hazard ratio

## Discussion

This is the first published study that has summarised treatment outcomes from a cohort of HIV negative and culture-confirmed MDR-TB and XDR-TB patients in Hunan Province, China. The overall rate of treatment success was 57% (95% CI: 52%–61%). This result is consistent with reported global (52%) and national (56%) MDR-TB treatment success rates [2, 6], and with the MDR-TB treatment success rate recorded in another study conducted in patients from Chinese referral hospitals [17]. However, the rate is still far short of the WHO target of 75% treatment success, and lower than in other studies conducted in Pakistan [18], Switzerland [19], the United Kingdom [20], Ethiopia [21], and Portugal [22]. Low treatment success rates have been also reported in other studies conducted in China among similar patient groups (i.e. among those who are HIV-negative with culture-confirmed MDR-TB or XDR-TB) [23, 24]. This low rate of treatment success among MDR-TB patients poses a serious threat for national TB control efforts as patients may develop additional resistance and may also transmit drug resistant forms of TB to others [25, 26].

In our study, the prevalence of XDR-TB among all MDR-TB was 2% (95% CI: 1%-4%). This is a similar finding to a study conducted in Beijing that reported a prevalence of XDR-TB among all MDR-TB patients of 1.4% [17]. However, it is lower than the results from the national drug resistance survey, and other population-based and hospital-based studies from China, which have reported a prevalence of XDR-TB ranging from 8% to 12% among all MDR-TB patients [6, 23, 27, 28]. This may be due to the fact that our study subjects were HIV-uninfected. In a previous study, it was reported that HIV infection is a risk factor for XDR-TB [29]. The lower prevalence could be also due to differences in the setting and time period. Geographical variation has been reported in the occurrence of drug resistance TB in China [30]. The proportions of MDR- and XDR-TB among TB patients were higher in some provinces than the overall national prevalence [6, 12, 13].

The other important finding of our study was that just over one quarter (27%) of the patients were lost to follow-up. This was higher compared to a pooled rate of 13% identified in a systematic review, and with results from other studies conducted in South Africa (17% -20%), Malaysia (17%) and the United Kingdom (7.8%) [20]. It was also higher than results reported from Beijing (17.4%) [17], and other parts of China (5.46%) [23]. Loss to follow up mainly occurred during the continuation phase of treatment (i.e. after 6 months of treatment), unlike a study conducted in Ethiopia in which loss to follow-up occurred during the intensive phase of treatment [21]. The proportion of patients lost to follow-up contributes to the low treatment success rate. To solve this problem in Hunan Province, the hospital has designed a tracing mechanism whereby patients who are not yet enrolled on treatment and who miss the monthly follow-up appointment are notified to the Center for Disease Control and prevention (CDC) staff of the county where the patient resides. Then the CDC staff, with the assistance of the local health professionals, trace the patient and discuss with them the importance of continuing their treatment. A study of the reasons for loss to follow-up conducted in the Hunan Chest Hospital using this tracing mechanism, found that patients were lost due to: economic hardship; a misconception that they were cured when their symptoms resolved or when they were informed of their culture conversion result from positive to negative; death – that was not reported to the TB programme or the hospital; and emigration to another area for work [31]. Adopting and implementing the shorter MDR-TB regimen (i.e. with a treatment duration of 9–12 months), recently recommended by WHO, might be a solution for reducing the proportion of patients lost to follow up, especially for patients who interrupt their treatment as a result of outwards-migration and economic hardship [32].

Additionally, a high rate of treatment failure was observed in our study. Almost all MDR-TB patients who had the outcome of treatment failure were sputum smear positive at the start of treatment. Presumably, these patients remained sputum smear positive throughout treatment. This ongoing infectiousness might contribute to a serious epidemic of drug-resistant TB in China [6], including primary transmission of MDR-TB and XDR-TB [33]. Recent studies have shown that high number of cases with MDR-TB and XDR-TB occurred due to primary transmission [7, 8, 34].

The number of patients diagnosed with MDR-TB and started on treatment has increased over time. Treatment outcomes have also improved over time. The proportion of patients who experienced poor treatment outcomes was higher in patients who started treatment during 2011 and 2012 than patients who started treatment during 2014. This might indicate improvements in care over time, including improved diagnostic services, treatment and follow up. Since 2013, the hospital has introduced new molecular diagnostic methods such as line probe assays and Xpert® MTB/RIF, that shorten the diagnostic period. Additionally, with the support of funds from the Global Fund to fight AIDS, TB and Malaria, new anti-TB agents such as capreomycin and cycloserine were used for the treatment of MDR-TB for the first time in 2013. Meanwhile, a tracing mechanism was implemented to support follow up of patients during this period.

We also found that resistance to ofloxacin was an independent predictor of poor treatment outcomes, which concurs with previous studies showing that baseline ofloxacin resistance was associated with poor MDR-TB treatment outcomes [35–38]. This finding suggests the importance of fluoroquinolones, especially ofloxacin in MDR-TB treatment. Baseline DST for fluoroquinolones will ensure appropriate administration of an effective regimen for MDR-TB patients.

This study has some limitations. Firstly, since the study was based on secondary data, some important variables such as types of anti-TB drug prescribed to the patients; side effects of the drugs; haematological data such as hemoglobin and liver function test results; and co-morbidities were not available in the registers and therefore were not included in our study. Secondly, the small number of patients with XDR-TB may have limited our ability to comprehensively assess treatment outcomes and predictors of poor treatment outcomes. Thirdly, since all HIV and TB co-infected patients in China are referred to HIV-specialized hospitals and not admitted to Chest Hospitals, our study has not assessed the impact of HIV infection on the treatment outcomes of MDR-TB and XDR-TB patients. Previous studies have shown that in countries where patients with XDR-TB also had HIV co-infection, extremely high mortality rates have been reported [23, 39–43].

## Conclusions

The present study showed sub-optimal treatment success rates among patients with MDR-TB and XDR-TB, and high rates of loss to follow-up in Hunan Province, China. However, the study has provided some evidence that treatment success rates have increased over time. Resistance to ofloxacin was an independent predictor of poor treatment outcomes. Strategies should be designed to reduce the high rate of poor treatment outcomes and loss to follow-up.

## Abbreviations

AHR: Adjusted hazard ratio
CDC: Center for disease control and prevention
CHR: Crude hazard ratio
CI: Confidence interval
DOT: Directly observed therapy
DST: Drug susceptibility testing
HIV: Human immunodeficiency virus
HR: Hazard ratio
IQR: Interquartile range
MDR-TB: Multidrug-resistant tuberculosis
SD: Standard deviation
TB: Tuberculosis
WHO: World Health Organization
XDR-TB: Extensively drug-resistant tuberculosis
